# Combinatorial *ab initio* calculations and core spectroscopy unravel the electronic structure of nickel cobalt manganese oxide

**DOI:** 10.1038/s41598-025-89283-8

**Published:** 2025-02-17

**Authors:** Timo Reents, Elmar Kataev, Daniel Duarte-Ruiz, Regan G. Wilks, Raul Garcia-Diez, Marcus Bär, Caterina Cocchi

**Affiliations:** 1 Institute of Physics, Carl von Ossietzky Universität Oldenburg, Carl-von-Ossietzky Strasse 9, 26129 Oldenburg, Germany; 2 Department of Interface Design, Helmholtz-Zentrum Berlin für Materialien und Energie GmbH (HZB), Albert-Einstein Str. 15, 12489 Berlin, Germany; 3 Energy Materials In-Situ Laboratory Berlin (EMIL), HZB, Albert-Einstein Str. 15, 12489 Berlin, Germany; 4 Department of Chemistry and Pharmacy, Friedrich-Alexander-Universität Erlangen-Nürnberg (FAU), Egerlandstr. 3, 91058 Erlangen, Germany; 5 Dept. X-ray Spectroscopy at Interfaces of Thin Films, Helmholtz-Institute Erlangen-Nürnberg for Renewable Energy (HIERN), Albert-Einstein-Str. 15, 12489 Berlin, Germany; 6 Center for Nanoscale Dynamics, Carl von Ossietzky Universität Oldenburg, Carl-von-Ossietzky Strasse 9, 26129 Oldenburg, Germany

**Keywords:** Condensed-matter physics, Materials science

## Abstract

The rising interest in complex oxides for energy storage applications calls for the development of efficient computational schemes that enable exploring the vast configurational space of these materials to guide and complement experiments. In this work, we adopt a high-throughput screening method based on density-functional theory to investigate the electronic-structure fingerprints of a specific stoichiometry of lithiated manganese-cobalt-nickel oxide, $\hbox {LiNi}_{0.8}\hbox {Co}_{0.1}\hbox {Mn}_{0.1}\hbox {O}_{2}$, which are relevant for the identification of the material in X-ray spectroscopy experiments. After creating the candidate structures in an automated fashion, we inspect their structural characteristics and electronic properties focusing specifically on the Ni and O contributions to the density of states. To do so, we exploit data analysis schemes that provide us with a metric to classify the considered structures according to the properties of interest, including the oxidation state. Comparison with X-ray absorption spectroscopy measurements confirms the robustness of the developed computational approach and reveals the most likely composition of the probed sample.

## Introduction

Complex transition-metal oxides are among the most promising material classes for applications in lithium-ion batteries^1,2^. Lithiated nickel-cobalt-manganese oxides, often referred to as NCM (chemical formula: $\hbox {LiNi}_{x}\hbox {Co}_{y}\hbox {Mn}_{z}\hbox {O}_{2}$), are key materials for this technology, as they offer a compelling combination of high energy density^3^, which is vital for longer battery life, and potentially lower costs compared to other cathode materials^4^. As the demand for electric vehicles and portable electronic devices continues to surge, NCM cathodes are being actively researched to address the critical challenge of increasing energy storage capacity^5–9^. The compound $\hbox {LiNi}_{0.8}\hbox {Co}_{0.1}\hbox {Mn}_{0.1}\hbox {O}_{2}$ (NCM-811) is a frontrunner in this field. Thanks to its high nickel content, it offers wide potential to increase energy storage capacity compared to other stoichiometries^10,11^. However, these advantages come with a trade-off: similar to other Ni-rich compounds, NCM-811 has stability issues^12–15^ particularly at high degrees of delithiation^16–18^ and tends to overheat more than other NCM formulations^19^. Nonetheless, NCM-811 remains under the limelight of both fundamental and applied research on energy storage materials^20–24^.

The structural complexity of NCM-811 represents a major challenge in the study of this compound. The coexistence of three transition-metal elements and the diffusing lithium ions in operational conditions give rise to non-trivial atomic arrangements in the layered oxide structure which are detrimental to the material stability^25,26^. Gaining insight into the structure-property relationships of NCM-811 is thus essential for its application in long-life devices. This goal can be achieved with joint experimental and theoretical efforts. X-ray absorption spectroscopy (XAS)^27^ is an established technique to study the electronic structure of complex materials such as layered transition metal oxides^28–30^. It provides element-specific information about oxidation states and local structure including atomic coordination^31^. From a theoretical perspective, *ab initio* calculations represent the most powerful approach to investigating and predicting the structural and electronic properties of complex materials on a fundamental level. While corresponding methods like density-functional theory (DFT) allow for material simulations without relying on empirical parameters, they necessitate the knowledge of the sample’s structure and chemical composition. In NCM-811, where the atomic arrangement is not uniquely defined, the ability of *ab initio* approaches to complement experimental research is severely hindered. The recent advent of data-driven methods has largely mitigated this problem.

Computational and experimental material databases are invaluable resources for exploring structural and chemical configurations and offer an irreplaceable starting point for high-throughput screening, machine learning, and related schemes^32,33^. As such, they enable the exploration of vast configurational spaces and thus the simulation of material properties approaching a realistic level of complexity. Such predictive power is not restricted to identifying stable compositions or other characteristics related to the ground states of the investigated systems but can be extended to spectroscopic properties, too. For example, artificial intelligence models applied to XAS enable the interpretation of the spectra with unprecedented accuracy and efficiency^34,35^. These advances demonstrate the potential of combining state-of-the-art computational modeling and spectroscopy techniques to identify and decipher the signatures of complex materials.

Despite the aforementioned advances based on data-driven methods, characterizing the electronic structure and spectroscopic properties of complex materials like NCM-811 using both experimental and theoretical methods remains a challenge. Particularly intricate is the connection between the spectral fingerprints exhibited by lithiated and delithiated samples and their degradation products and the electronic structure of the materials. For this purpose, the development of new computational techniques that enable deciphering the signatures of specific compounds and the local atomic environment therein is essential and can significantly contribute to a deeper understanding of the samples in view of ultimately improving their energy storage performance, for example through the modulation of their chemical composition including the introduction of dopants.

Motivated by these pressing questions, in this work, we develop and apply an efficient scheme for high-throughput calculations based on DFT to predict and analyze the electronic-structure fingerprints of NCM-811 in combination with XAS experiments. Starting from database mining, we generate a comprehensive set of candidate structures encompassing all possible atomic arrangements arising from the occupation of lattice sites by the transition metal atoms. We deal with the intrinsic spin-polarization of these species using an in-house implemented procedure that automatically creates initial magnetic configurations and filters the most stable ones (see Sec. II B). For the latter, we compute the atom-projected density of states (PDOS) accounting for the contributions in all crystal sites. We analyze this large amount of data by adopting hierarchical clustering models already tested on spectral data^36,37^ in order to disclose correlations between structural and electronic properties. We focus on the local environment of the atomic species to understand its impact on the electronic structure of the materials; in the case of nickel, we inspect in detail the effects of different oxidation states^38,39^. The outcomes of this analysis provide a comprehensive understanding of the local atomic and electronic characteristics of NCM-811 and represent the baseline to identify the key structural features that govern Li-ion diffusion and other critical properties of these cathode materials. This knowledge is relevant for their investigation in *operando* conditions and for the design of optimized compounds with enhanced energy-conversion and -storage performance.

## Computational methods

**Fig. 1:**
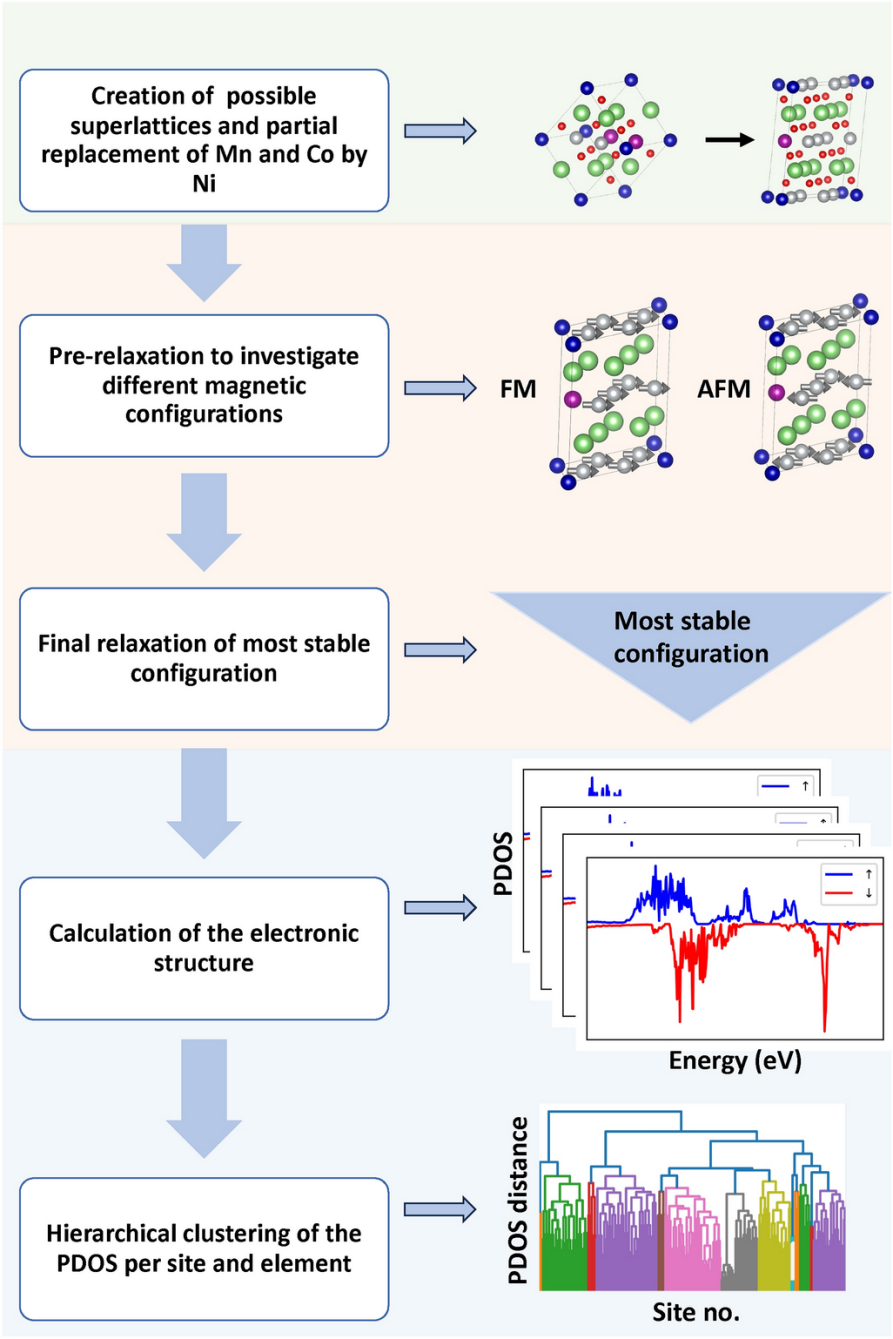
Flowchart of the computational procedure adopted in this work with a schematic view of each main step. The green area shows the generation process of structural candidates, the orange area covers the optimization workflow, and the blue area corresponds to the analysis of the PDOS.

The computational procedure adopted in this work is visually summarized in Fig. 1. In the following subsections, we describe each step in detail.

### Structure generation

The first step of our study consists of the generation of the candidate structures (Fig. 1, top panel in green). In this process, we start from $\hbox {Li}_5\hbox {Mn}_2\hbox {Co}(\hbox {NiO}_5)_2$ available in the Materials Project database^40^ (entry-id: mp-1222667; database v2022.10.28) to create the desired stoichiometry $\hbox {LiNi}_{0.8}\hbox {Co}_{0.1}\hbox {Mn}_{0.1}\hbox {O}_{2}$, and we apply subsequently an enumeration algorithm as implemented in the enumlib library^41^. To this end, we consider supercells twice as large as the initial unit cell of $\hbox {Li}_5\hbox {Mn}_2\hbox {Co}(\hbox {NiO}_5)_2$ and allow Mn and Co sites to be populated by Ni while keeping the concentration fixed to 8 Ni atoms, 1 Mn atom, and 1 Co atom as in NCM-811^42,43^. Under these constraints, we obtain 28 crystal structures containing 40 atoms each on 7 distinct superlattices that we investigate in detail in the following analysis.

### High-throughput DFT calculations

After generating the structural candidates, we proceed with the *ab initio* calculations consisting of a pre-relaxation stage and the final structural optimization (Fig. 1, middle panel in orange). All runs are performed using DFT^44,45^ as implemented in the plane-wave code Quantum ESPRESSO, version 7.0^46,47^. Supported by tests performed on rock-salt CoO in the preliminary stage of this study and by the existing literature^48–50^, we adopted ONCV SG15 pseudopotentials^51,52^. Furthermore, we approximated the exchange-correlation potential of DFT with the Perdew-Burke-Ernzerhof (PBE) implementation of the generalized gradient approximation. Despite the known limitations of this functional in estimating band gaps of solids in general and transition-metal oxides in particular, we purposely stick to it in the present analysis due to the missing consensus regarding the most effective way to cure its issues. Including a Hubbard correction term *U* is a common practice to lift this problem in transition-metal oxides^53,54^. However, the choice of a suitable value for *U* is not obvious in a complex material like NCM-811^21,55^. In particular, it is still under debate^23,56^ whether a single *U* can be adopted for structures characterized by different local environments. Since energies calculated with different Hubbard-*U* values are not directly comparable^57^, such an issue would invalidate the present high-throughput screening analysis. Additional tests performed on CoO using the SCAN functional^58^ in the preliminary stage of this work did not lead to any significant improvement of the PBE results in the description of the conduction region of the material, which is most relevant in the interpretation of the fingerprints from XAS. For this reason, we stick with PBE in analogy with previous work^21,23^. Finally, despite the relatively large size of the transition-metal species in the considered compounds, we did not include spin-orbit coupling in our DFT calculations, because the induced splitting is significantly smaller than the resolution of the measurements, both instrumental and due to lifetime broadening. Thus, corresponding results would not directly compare to experimental data and, as such, would not contribute to the core analysis of this work.

The workflow developed for this work is built on top of the AiiDA plugin for Quantum ESPRESSO^59,60^. The existing routines are locally modified and extended to handle the different magnetic configurations that arise in generating structures containing three transition-metal species. To explore the magnetic ground state, we used enumlib^41^ to generate distinct spin configurations on the available atomic sites following the approach proposed by Horton et al.^61^. We considered initial configurations with non-magnetic, antiferromagnetic, and ferromagnetic orderings. As the ferromagnetic arrangement proved to be the energetically most favorable one, subsequent DFT calculations were initialized in this spin configuration.

To efficiently screen the generated configurations, a pre-relaxation step is performed with the following parameters and convergence criteria (see Refs.^46,47^ for their meaning and default values): a homogeneous **k**-mesh with a spacing of 0.6 Å^-1^, a force threshold of $10^{-3}$ Ry/bohr, and an energy cutoff of $10^{-4}$ Ry. The resulting most stable structures are further relaxed with tighter force and energy thresholds of $2\cdot 10^{-4}$ Ry/bohr and $10^{-5}$ Ry/atom, respectively, and with a uniform **k**-mesh with a spacing reduced to 0.2 Å^-1^ for Brillouin-zone sampling. For the PDOS calculations conducted on the 28 stable structures, a finer **k**-grid with a spacing of 0.1 Å^-1^ is employed together with wave-function and charge-density cutoffs of 110 Ry and 440 Ry, respectively.

### PDOS clustering analysis

The final stage of the workflow includes the analysis of the site-resolved PDOS (Fig. 1, bottom panel in blue). To accomplish this task, the metric proposed by Kuban et al.^62^ is implemented and included in the workflow. The discretized PDOS fingerprints are initially calculated separately for both spin channels; subsequently, they are concatenated to obtain the final result used for the pairwise comparison of the site-resolved PDOS based on the Tanimoto coefficient^62^. To apply hierarchical clustering, the PDOS similarity metric is interpreted as a distance metric by inverting the scale so that a value of 0 corresponds to the minimal distance and vice versa. A pairwise distance matrix is constructed based on this distance metric representing the difference between the spin-polarized PDOS of two sites. The resulting distance matrix is used for hierarchical clustering of the sites into clusters exhibiting similar features and shapes in their PDOS. The complete-linkage method is used to interpret the distance between different clusters as the maximum distance of pairs across two clusters. The clustering process is carried out for all Ni sites and all O sites separately, analyzing only the elements occurring multiple times (apart from Li) and providing significant contributions to the PDOS to interpret X-ray spectroscopy measurements.

The grid applied to discretize the PDOS is uniform in the energy axis (*x*) and the one counting the number of states (*y*). A step of 0.05 eV is used in the *x*-direction while along *y*, it is determined by dividing the maximum value of the PDOS for all Ni- and O-sites into 350 equidistant bins. The energy range from -5 eV to 4 eV is taken into account to compare with edge features in resonant inelastic X-ray scattering (RIXS) measurements; the Fermi level and the valence band maximum are set to 0 eV in the case of metals and semiconductors, respectively. Finally, the number of clusters was chosen based on the resulting dendrogram of the hierarchical clustering procedure. For Ni, we did not only rely on the dendrogram but also combined it with the a posteriori knowledge that we can identify three different Ni oxidation states. Therefore, we favoured smaller number of clusters to enable a simpler relation between clusters and oxidation states. Based on the dendrogram, we selected four instead of three clusters. Otherwise, the variance within each cluster would get too large. In case of oxygen, we only used the information from the dendrogram to find an appropriate number of clusters and additionally verified that the maximum variation within each cluster is still small enough.

## Experimental methods

O K-edge RIXS maps were acquired at beamline 8.0.1 of the iRIXS endstation at the Advanced Light Source, Lawrence Berkeley National Laboratory. The compound $\hbox {LiNi}_{0.8}\hbox {Co}_{0.1}\hbox {Mn}_{0.1}\hbox {O}_2$, obtained from the company BASF, was mounted on a sample holder in the Ar-filled glovebox and transferred from the glovebox into the ultrahigh vacuum main chamber (base pressure lower than $5 \times 10^{-9}$ Pa) using a sealed sample transfer kit. The monochromator slits were set to 50/50, and the samples were continuously moved within a small region (2 mm) relative to the focused beam position to minimize beam irradiation-induced effects. The photon energy was calibrated using a reference $\hbox {TiO}_2$ sample^63^. The emission spectra at each excitation energy were collected for 90 s. The RIXS map was generated from the emission spectra^64^. The emission energy was calibrated based on the elastic peak line on the RIXS map. The partial fluorescence yield (PFY) X-ray absorption and emission spectra (XAS & XES) were obtained by integrating RIXS map in one direction over emission or excitation energies correspondingly. The Fermi level was approximated as the intersection of PFY XAS/XES and set to 0 eV.

## Results

### Structural analysis

**Fig. 2:**
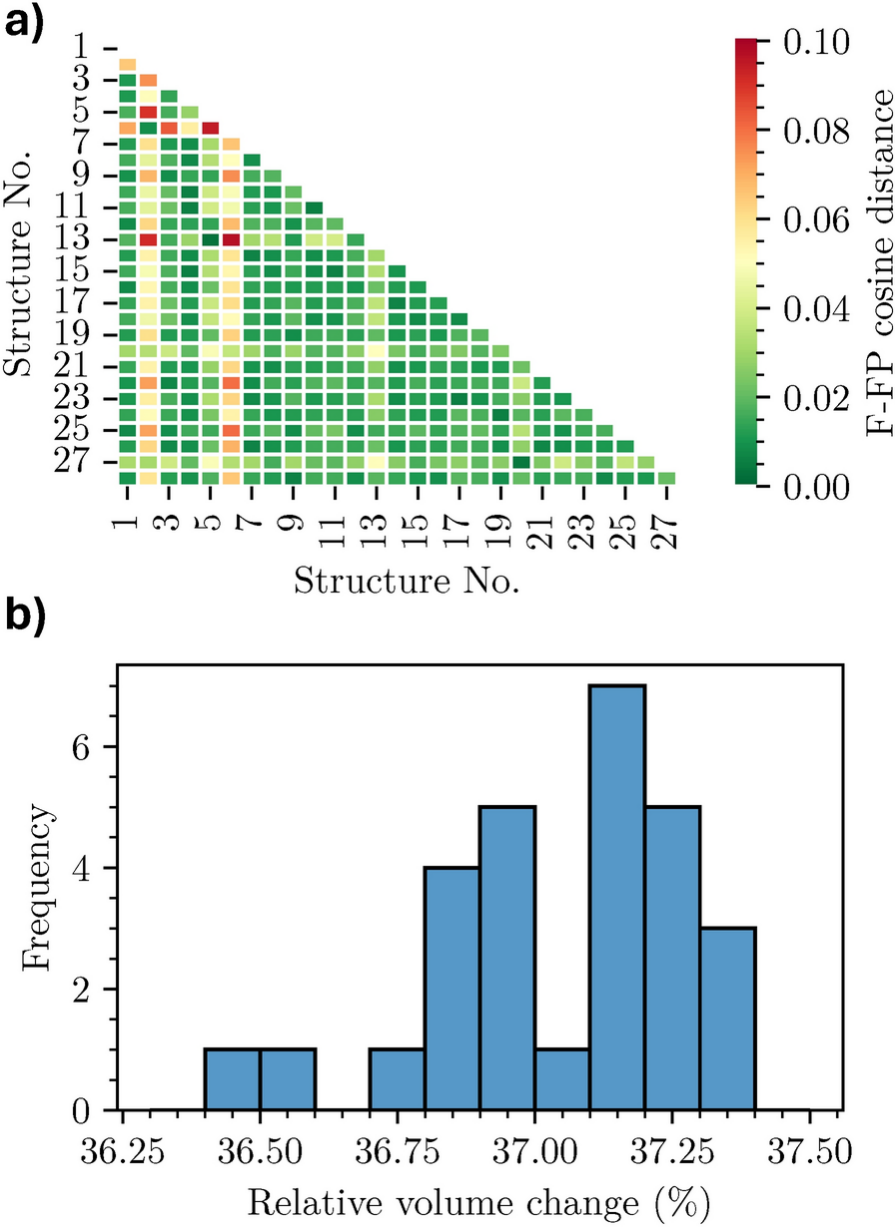
a) Pairwise similarity map between relaxed structures evaluated from the F-fingerprint and cosine distance. b) Relative volume change upon structural optimization in the considered structures: all investigated systems are included in the visualized region on the horizontal axis.

We start by analyzing the structural similarity of the generated structures using the F-fingerprint method proposed by Oganov et al.^65^, to which we refer for details about the implementation. This metric is based on the partial radial distribution function: a low value indicates high similarity and vice versa. The results obtained for the optimized structures are shown in Fig. 2a. The overall picture provided by this graph indicates large similarities among the considered systems except for some outliers that stand out. These differences arise during relaxation and are ascribed to the modification of the local environment occurring upon force minimization. For reference, an analogous graph for the initial structures is plotted in the Supporting Information (SI) Fig. S1, showing variations that are orders of magnitude smaller.

To quantify the structural modifications induced by DFT optimization, we visualize in Fig. 2b the relative change of the unit cell volume during relaxation. We specify that reported data are referred to the 28 NCM-811 crystal structures generated by us with the enumeration procedure described in Sec. II A before and after DFT optimization. The obtained variations are on the order of 37% for all examined structures. This result confirms the expected large variations due to the perturbation of the local environments. The relative volume change differing by approximately 1% emphasizes the importance of optimizing each structure rather than relying on the pre-optimization available from the databases or performed in the initial steps of our workflow. In terms of energetic stability, the maximum energy difference with respect to the most stable structure is approximately 8 meV/atom (further details in Table S1), which is below the thermal contribution at room temperature of approximately 25 meV. Therefore, none of the structures is significantly favored over the others. These findings are further supported by the results by Sun et al.^66^, where an even broader set of structural arrangements of the transition-metal sites was studied and energy differences smaller than 10 meV/atom were obtained with respect to the minimum energy configuration for most of the structures.

**Fig. 3:**
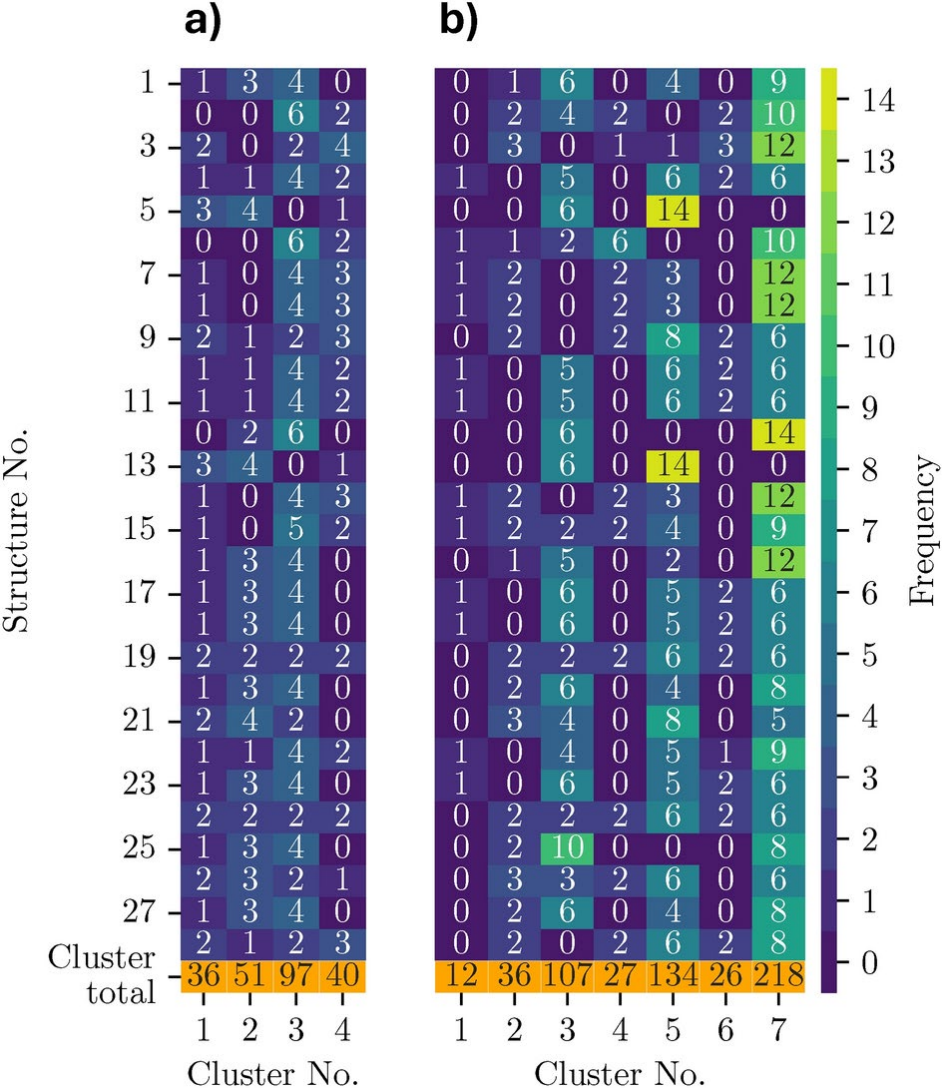
Distribution of the (**a**) Ni and (**b**) O sites of each structure over the different PDOS clusters. The reported values indicate the occurrence of Ni (panel a) and O atoms (panel b) in the clusters of each structure while the color shades mark their frequency in the clusters. The bottom orange rows represent the total number of sites per cluster.

### PDOS clustering

**Fig. 4:**
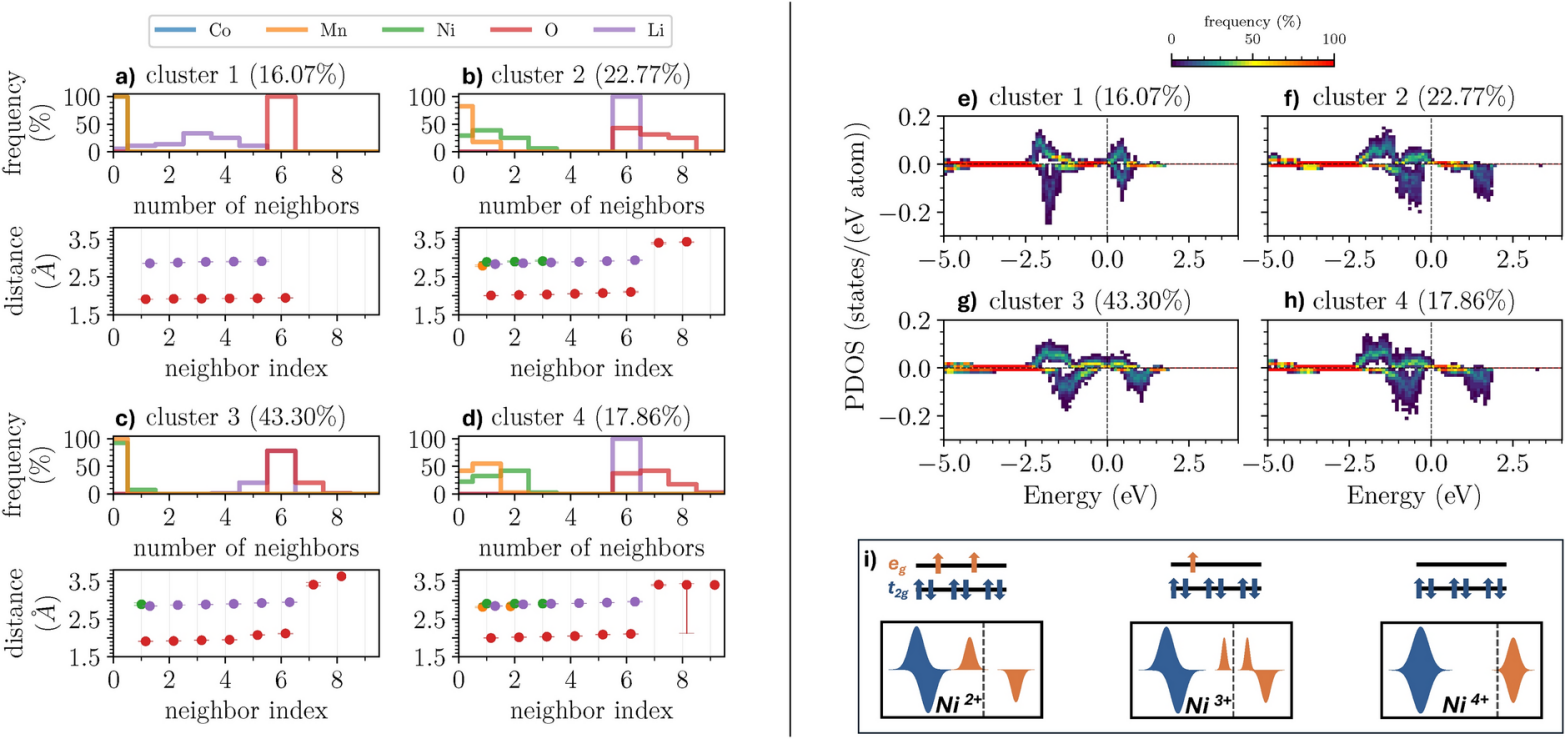
(**a**-**d**) Structural properties of the considered NCM-811 clusters in the vicinity of the Ni atoms with their relative amount indicated in parenthesis. The plots in the upper panels show the number of atoms of a given species in the local environment of the corresponding Ni-site. The lower panels represent the median of the distances to a given element including the 95%-confidence interval calculated via bootstrapping but hardly visible due to the small variance. The distances are plotted over the neighbor-index (counting the ordered neighbors per species) whereby the index 1 corresponds to the closest neighbor of a species. The data points are arbitrarily displaced around the actual integer values (indicated by the gray lines) to enhance visualization. (**e**-**h**) Clustered PDOS for the Ni sites of the 28 structural candidates of NCM-811. Each site is considered separately whereby the spin-polarization is taken into account during the clustering (positive and negative values are associated with spin-up and spin-down contributions, respectively, and the Fermi energy is set to zero). The PDOS in each cluster is represented as a 2D-histogram to allow statistical conclusions (the counts per cell are normalized by the number of sites per cluster so that each column sums up to 100% per spin channel) whereby the x-axis is discretized in steps of 0.05 eV. (**i**) Schematic representation of the electronic configuration across the $\hbox {Ni-t}_{2g}$ and $\hbox {Ni-e}_g$ orbitals (top) and the ideal PDOS structure (bottom) for different Ni oxidation states.

We partition the PDOS with the contributions from the Ni-sites and O-sites into clusters based on the similarity of their electronic structure. These results will be discussed in detail in the context of Fig. 4e-h and Fig. 6g-m. To understand whether specific clusters are associated with particular structures or if all structures contribute to each cluster, we analyze the distribution of Ni and O sites from different structures across these clusters. For this purpose, we partition the relaxed configurations and count the number of Ni and O sites assigned to each cluster, see Fig. 3, where each row represents a different structure, and each column represents a PDOS cluster. The color bar indicates the number of sites from that structure that belong to the corresponding cluster.

In Fig. 3a, we notice a preference for Ni distribution across multiple clusters with most structures exhibiting at least one or two Ni atoms in each cluster. Clusters 2 and 3 stand out for their higher concentration of Ni with three or even more sites. Conversely, cluster 4 typically accommodates one or two Ni atoms, and even lacks Ni entirely. Cluster 1 predominantly contains one single Ni site. Only a few structures possess clusters with five or six Ni atoms, and none have more than six.

The distribution of O sites shown in Fig. 3b is qualitatively very different. In this case, 7 clusters are identified to host the 20 O atoms included in the constructed supercells. There is a clear predominance in all structures of the occupation of a few clusters with most O atoms (cluster 3, 5, and especially 7). In reverse, the remaining clusters host a maximum of one (cluster 1) or a maximum of three (clusters 2, 4, 6) O sites. We recall that this analysis refers to the clusters extracted from the simulated NCM-811 structures and not to the structures themselves. As such, the relative abundance or scarcity of Ni and O atoms in the clusters does not directly influence the composition of the cathode material.

### Structural and electronic properties of Ni clusters

We continue with the analysis of the structural and electronic properties of the Ni-sites within the different structures based on the cluster partition presented above, see Fig. 4a-d, where Voronoi tessellation is used to determine the local environments of the sites. When considering neighboring sites and their distances, we limit our analysis to those that directly share a Voronoi facet with the investigated site, constituting its local environment. The upper panel of each subplot displays the number of neighboring sites of a given species in the local environment of each Ni-site within the cluster. It is important to note that the number of neighbors is always reported from the perspective of the local environments of Ni. For example, with “Mn” we refer to the relative amount of Ni sites in each cluster with a given number of Mn-sites in their local environment and not to the number of Mn sites per cluster. In the bottom panels, the distances between the considered Ni-site and the neighboring sites (sorted by ascending distance) in its local environment are shown. All properties are averaged within each cluster: the distances are given as the median value, and the number of neighbors is given as a relative amount, e.g., in cluster 1, 100% of the Ni sites do not have neighboring Mn sites in their local environment.

The number of neighboring sites and distances shown in Fig. 4a-d is reported in Tables 1 and 2, respectively. Cluster 1 shows a highly symmetric and ordered environment with minimal variation in the distances (around 1.9 Å for O and 2.9 Å for Li). In contrast, clusters 2 and 4 are more varied with slightly longer distances to neighboring sites (around 2.05 Å). The number of Li neighbors shows a smaller spread compared to cluster 1. Moreover, these two clusters are the only ones that show a significant amount of adjacent Ni sites in the local environment. In cluster 4, 55% of the local environments of the Ni sites exhibit one neighboring Mn site, too. Finally, clusters 2 and 4 show additional O-neighbors, e.g. 7 or 8 O-neighbors in total, in contrast to the 6 found for all Ni sites in cluster 1. Cluster 3 shows an identical local environment as cluster 1. Furthermore, while the first four Ni-O distances are comparable to the shorter ones in cluster 1, the 5th and 6th nearest O-neighbors show a larger separation, comparable to the ones found in clusters 2 and 4. Apart from that, similar to clusters 2 and 4, around 20% of the Ni-sites contain 7 O-neighbors, whereas the 7th is significantly farther away, most likely reducing the impact on the Ni-site. Overall, the most significant distinctions between the clusters arise from variations in the local environments, specifically the presence or absence of additional transition metal sites and differences in Ni-O bond lengths. Notably, while these inter-cluster differences are pronounced, intra-cluster similarities are striking, validating the effectiveness of our clustering approach.

In Fig. 4e-h, we report the spin-resolved PDOS calculated from the contributions of the Ni sites in the considered clusters. All plots feature two peaks in each spin channel. In the case of cluster 1 (Fig. 4e), both channels are nearly aligned, suggesting negligible magnetic effects. Furthermore, in this cluster, there is the largest energetic separation between the peaks in the spin-up channel, while in all clusters, the Ni-contributions with spin-down are very similar. In the case of clusters 2 and 4 (Fig. 4f and h), the two peaks in the spin-up channel are separated by a very small gap of the order of a few hundreds of meV. On the other hand, cluster 3 (Fig. 4g) features a single peak between -2 and -1 eV with very weak maxima in the region from -1 to 1 eV.

The previous analysis allows us to compare the structural and electronic features of the identified clusters. While the number of neighbors has a limited impact on the PDOS, contrary to expectations, the presence or absence of Mn atoms significantly influences the electronic structure. The near-constant distribution of Li distances suggests a minimal contribution to the observed variations. Therefore, the strong hybridization between transition-metal 3*d* and O 2*p* orbitals primarily drives the electronic structure differences.

**Table 1:** Number of neighbors of a given species in the local environments of the Ni-sites. All results are normalized per neighboring element and cluster.

Element	Cluster occurrences	1	2	3	4
Li	0	0.056	-	-	-
	1	0.111	-	-	-
	2	0.139	-	-	-
	3	0.333	-	-	-
	4	0.250	-	0.010	-
	5	0.111	-	0.206	-
	6	-	1.000	0.784	1.000
Mn	0	1.000	0.824	1.000	0.425
	1	-	0.176	-	0.550
	2	-	-	-	0.025
Ni	0	1.000	0.294	0.928	0.225
	1	-	0.392	0.072	0.325
	2	-	0.255	-	0.425
	3	-	0.059	-	0.025
O	6	1.000	0.431	0.784	0.375
	7	-	0.314	0.206	0.425
	8	-	0.255	0.010	0.175
	9	-	-	-	0.025

**Table 2:** Median distances to the neighboring sites in the local environment of the Ni-sites. *element* indicates the species of the neighboring site and *index* represents the index of the sorted (in ascending order) neighboring sites per species. All results are given in Å and aggregated per neighboring element and cluster.

Element	Cluster neighbor index	1	2	3	4
Li	1	2.86	2.84	2.84	2.85
	2	2.88	2.86	2.87	2.89
	3	2.90	2.88	2.89	2.91
	4	2.91	2.90	2.90	2.92
	5	2.91	2.92	2.93	2.94
	6	-	2.94	2.95	2.96
Mn	1	-	2.80	-	2.82
	2	-	-	-	2.84
Ni	1	-	2.90	2.89	2.91
	2	-	2.90	-	2.91
	3	-	2.92	-	2.91
O	1	1.91	2.01	1.91	2.00
	2	1.92	2.02	1.92	2.02
	3	1.92	2.03	1.94	2.03
	4	1.93	2.05	1.95	2.05
	5	1.93	2.07	2.08	2.09
	6	1.94	2.10	2.12	2.11
	7	-	3.40	3.41	3.41
	8	-	3.43	3.63	3.41
	9	-	-	-	3.40

**Fig. 5:**
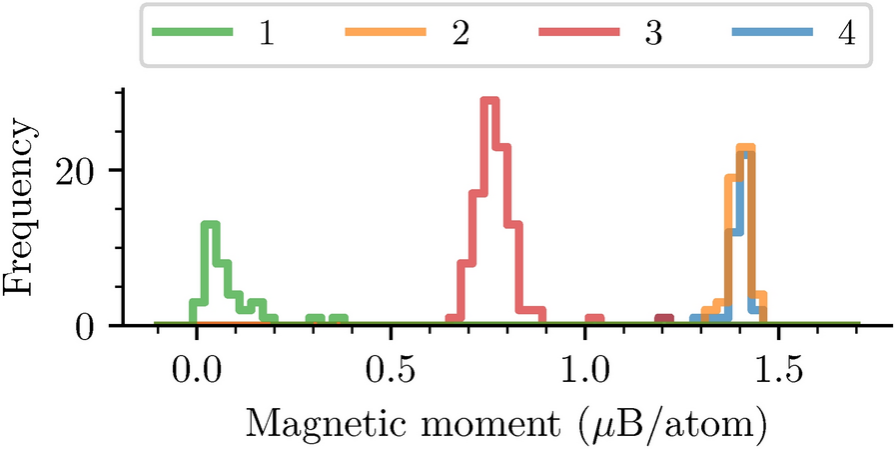
Distribution of the magnetic moments per Ni site across all structures. The color code marks the four Ni clusters.

The discussion above and the visual inspection of Fig. 4e-h suggest the shift between the two spin channels as a striking indicator to differentiate among the clusters. To deepen our analysis in this direction, we report in Fig. 4i the electronic configuration and the expected PDOS in the presence of different Ni oxidation states. The magnetic moments per cluster, as depicted in Fig. 5, can be linked to specific Ni oxidation states^23^. This allows us to utilize clusters as representative entities, providing insights into the underlying structural and electronic characteristics of different Ni oxidation states.

Cluster 1 shows the smallest magnetic moments (approximately $0.1\,\mu _{\textrm{B}}$) corresponding to the $\hbox {Ni}^{4+}$ oxidation state with no unpaired electrons in the $\hbox {Ni-e}_g$ orbitals, in agreement with the symmetry of the two spin channels. We note in passing that this oxidation state is unstable and thus, NCM-811 samples with $\hbox {Ni}^{4+}$ do not exist. Cluster 3 shows intermediate magnetic moments on the order of $0.8\,\mu _{\textrm{B}}$: hence, this cluster can be related to the $\hbox {Ni}^{3+}$ oxidation state, exhibiting one unpaired electron in the $\hbox {Ni-e}_g$ orbitals. Clusters 2 and 4, characterized by the large structural similarities discussed above, are also similar in terms of the magnetic moments. Both clusters show the highest magnetic moments ($1.4\,\mu _{\textrm{B}}$) pointing to an oxidation state of $\hbox {Ni}^{2+}$ with two unpaired electrons. This analysis is confirmed by the visual similarities of the PDOS computed for the four clusters (Fig. 4e-h) and the schemes in Fig. 4i.

The magnetic moments reported in Fig. 5 are in very good agreement with the work by Dixit et al.^23^, revealing an average absolute magnetization of $1.4\,\mu _{\textrm{B}}$, $0.8\,\mu _{\textrm{B}}$ and $0.1\,\mu _{\textrm{B}}$ for the $\hbox {Ni}^{2+}$, $\hbox {Ni}^{3+}$, and $\hbox {Ni}^{4+}$ oxidation state, respectively (we compare the absolute magnetization since an antiferromagnetic configuration was considered in Ref.^23^). Our results are also in agreement with those of Yousuf et al.^67^, who obtained similar values for the slightly different Ni-rich stoichiometries $\hbox {LiNi}_{0.875}\hbox {Mn}_{0.125}\hbox {O}_2$ and $\hbox {LiNi}_{0.75}\hbox {Mn}_{0.25}\hbox {O}_2$ using DFT+U. Finally, the work by Liang et al.^68^ reports slightly larger ranges for Ni magnetic moments compared to our findings. This discrepancy might be attributed to the inclusion of a Hubbard U correction in their DFT calculations. Nevertheless, our results maintain the same trend and correlation between different oxidation states. Unfortunately, it is not possible to directly compare the magnetic moments reported in Fig. 5 with the experimental literature due to the lack of corresponding values for NCM-811 and related materials.

The presented method reveals additional structural features within the different local environments. Cluster 1 (Fig. 4a), having the highest oxidation state, possesses a well-defined number of O sites in its local environment with a nearly constant distance of around 1.9 Å in an octahedral arrangement. This distance is the smallest observed across all local environments, consistent with previous findings^69,70^. Similarly, clusters 2 and 4 (Fig. 4b and d) associated with $\hbox {Ni}^{3+}$ are characterized by nearby Ni sites. The number of these Ni sites (two or three) seems less important than their overall presence. Due to this local abundance of Ni, electrons are distributed among multiple Ni sites when interacting with the more electronegative O atoms. This results in a lower formal oxidation state compared to cluster 1. Cluster 3 (Fig. 4c), unlike clusters 2 and 4, closely resembles cluster 1 in its local environment. Primarily composed of O and Li sites, it also features Ni sites with four nearby O sites at similar distances to those in cluster 1. However, neighboring O sites with indices 5 and 6 exhibit significantly larger distances, similar to those in clusters 2 and 4. This suggests an elongation of the octahedral arrangement observed in cluster 1 (associated with $\hbox {Ni}^{4+}$) due to Jahn-Teller distortion^66^. The latter effect lifts the degeneracy of the $\hbox {Ni-e}_g$ orbital containing a single unpaired electron. Notably, $\hbox {Ni}^{3+}$ is the only Jahn-Teller active site among the Ni-clusters because it is the only one with unpaired electrons in the $\hbox {e}_g$ orbitals. The observed distances of 2.1 Å for the distorted bonds are in good agreement with previous work^69,71^. This analysis demonstrates that the presented method is useful for identifying different oxidation states^23^ and enables their direct correlation with the structural features.

**Fig. 6:**
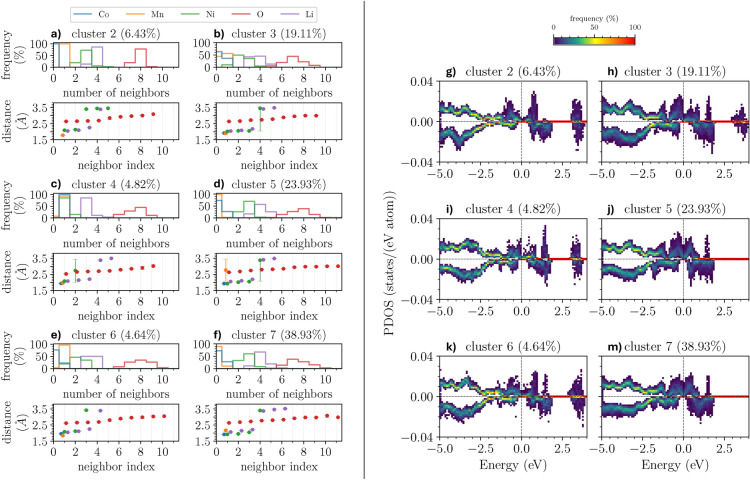
(**a**-**f**) Structural properties of the considered NCM-811 clusters in the vicinity of the O atoms. In the upper panels, the number of atoms of a given species in the local environment of the corresponding O-site is shown. The lower panels represent the median of the distances to a given element including the 95%-confidence interval calculated via bootstrapping (hardly visible due to the small variance). The distances are plotted over the neighbor-index (counting the ordered neighbors per species) whereby the index 1 corresponds to the closest neighbor of a species. The data points are arbitrarily displaced around the actual integer values (indicated by the gray vertical bars) to enhance visualization. The cluster number and the relative amount of O atoms per cluster are reported in the panel headers. Only clusters with an O contribution larger than 4% are shown. (g-m) Clustered PDOS for the O sites of the 28 NCM-811 structures. Each site is considered separately whereby the spin-polarization was taken into account during the clustering (positive and negative values are associated with spin-up and spin-down contributions, respectively, and the Fermi energy is set to zero). The PDOS in each cluster is represented as a 2D-histogram to allow statistical conclusions (the counts per cell are normalized by the number of sites per cluster so that each column sums up to 100% per spin channel) whereby the x-axis is discretized in steps of 0.05 eV. The cluster number and the relative amount of O atoms per cluster are reported in the panel headers.

### Structural and electronic properties of O clusters

In this section, we examine the oxygen contributions to the PDOS across various clusters, focusing on both the number of neighboring atoms and their distances, see Fig. 6 and Tables 3 and 4. Of the 560 O-sites analyzed, only clusters with significant site representation are included (cluster 1 is discussed in the SI). The structural properties of each cluster reveal that, while the number of neighboring atoms (Ni, Mn, Co, Li, and O) varies within each cluster, the distances between them serve as a more reliable descriptor for distinguishing the clusters. For example, in cluster 2, the number of Ni-neighbors ranges from 2 to 5, with a peak at 3, while the O-O distances vary between 2.7 and 3.1 Å. Cluster 3 has a broader distribution of O-neighbors (5 to 9) and similar Ni-neighbor counts, but the O-Mn distance is 1.9 Å, slightly larger than in cluster 2. Cluster 4 shows a similar trend in the number of O-neighbors, with O-Mn and O-Co distances of just under 2.0 Å and 1.95 Å, respectively. Clusters 5 and 7 stand out due to their smaller O-Ni distances (around 1.9 Å), whereas the O-O distances increase more uniformly compared to other clusters. In cluster 6, Mn is present throughout, and the number of O-neighbors ranges from 7 to 9, with O-Mn distances slightly smaller at 1.85 Å and O-Co distances around 1.95 Å. Across all clusters, the O-Li distances exhibit minimal variation, remaining around 2.1 Å. O-O separations are generally consistent but differ slightly in trends: clusters 2, 3, and 6 show a two-region distribution, while clusters 5 and 7 exhibit a more uniform increase. Overall, the variation in coordination numbers is significant within clusters, but interatomic distances - particularly O-Ni, O-Mn, and O-Co separations - provide clearer insights into the structural differences, echoing the trend observed in the earlier Ni cluster analysis.

**Table 3:** Number of neighbors of a given species in the local environments of the O-sites. All results are normalized per neighboring element and cluster.

Element	Cluster occurrences	2	3	4	5	6	7
Co	0	1.000	0.636	-	0.731	0.769	0.725
	1	-	0.364	1.000	0.269	0.231	0.275
Li	3	0.139	0.421	0.852	0.172	0.500	0.110
	4	0.861	0.449	0.111	0.567	0.500	0.674
	5	-	0.131	0.037	0.261	-	0.188
	6	-	-	-	-	-	0.028
Mn	0	-	0.439	0.074	0.985	0.038	0.899
	1	1.000	0.561	0.926	0.015	0.962	0.101
Ni	1	-	0.103	0.852	0.007	0.192	-
	2	0.194	0.495	0.148	0.261	0.462	0.326
	3	0.722	0.355	-	0.694	0.346	0.596
	4	0.056	0.047	-	0.037	-	0.078
	5	0.028	-	-	-	-	-
O	4	-	0.009	-	-	-	-
	5	-	0.056	-	0.022	-	0.014
	6	-	0.196	0.148	0.149	0.077	0.138
	7	0.194	0.439	0.296	0.269	0.269	0.381
	8	0.778	0.224	0.444	0.388	0.346	0.280
	9	0.028	0.075	0.111	0.149	0.269	0.151
	10	-	-	-	0.015	0.038	0.032
	11	-	-	-	0.007	-	0.005

**Table 4:** Median distances to the neighboring sites in the local environment of the O-sites. *element* indicates the species of the neighboring site and *index* represents the index of the sorted (in ascending order) neighboring sites per species. All results are given in Å and aggregated per neighboring element and cluster.

Element	Cluster index	2	3	4	5	6	7
Co	1	-	1.92	1.94	1.93	1.96	1.92
Li	1	2.05	2.04	2.08	2.06	2.05	2.07
	2	2.10	2.09	2.14	2.10	2.11	2.14
	3	2.26	2.18	2.20	2.19	2.25	2.21
	4	3.39	3.42	3.38	3.41	3.39	3.37
	5	-	3.49	3.49	3.49	-	3.46
	6	-	-	-	-	-	3.52
Mn	1	1.79	1.90	1.97	2.76	1.84	2.17
Ni	1	2.09	2.02	2.08	1.92	2.08	1.92
	2	2.13	2.06	2.73	2.01	2.12	1.95
	3	3.41	2.05	-	2.05	3.42	2.05
	4	3.45	3.46	-	3.38	-	3.40
	5	3.46	-	-	-	-	-
O	1	2.64	2.64	2.53	2.63	2.62	2.63
	2	2.66	2.66	2.65	2.68	2.65	2.68
	3	2.67	2.72	2.68	2.74	2.66	2.72
	4	2.69	2.74	2.70	2.79	2.69	2.77
	5	2.85	2.78	2.72	2.82	2.81	2.80
	6	2.93	2.89	2.79	2.85	2.90	2.83
	7	2.97	2.96	2.85	2.94	2.96	2.92
	8	3.01	3.00	2.90	2.98	2.98	2.97
	9	3.09	3.00	3.02	2.98	3.03	2.97
	10	-	-	-	3.00	3.05	3.08
	11	-	-	-	3.01	-	2.98

The O-PDOS shown in Fig. 6g-m is characterized by several sharp features that can be related to the structural properties discussed above. In general, all clusters exhibit a high similarity in the valence region for both spin channels while larger differences can be seen in the conduction region, mainly consisting of energetic shifts between the two spin channels. The region around the Fermi level, set to zero in Fig. 6g-m, is dominated by hybridized O- and Ni-states, as seen by contrasting the O-PDOS with the Ni-PDOS in Fig. 4e-h. Clusters 5 and 7, exhibiting smaller O-Ni distances, are characterized by a sharp oxygen peak in the PDOS around 0 eV, which increases with decreasing distance to the Ni-atoms. Such an inverse relationship is expected as the interaction and therefore the hybridization is stronger with shorter O-Ni separations. This outcome is further supported by the result obtained for cluster 3 (Fig. 6h) in comparison with clusters 2, 4, and 6 (Fig. 6g, i, k). Another characteristic peak between 1 and 2 eV is due to hybridized O- and Co-states. This assignment is confirmed by the absence of this maximum in the PDOS of cluster 2 (Fig. 6g), which does not contain any site with a neighboring Co atom. Cluster 6, which has a small number of sites with adjacent Co atoms, accordingly features only one weak peak between 1 and 2 eV (Fig. 6k). The maximum between 1 eV and 0 eV is also due to the presence of Co (see Fig. S6), as shown for some sites in cluster 3 (Fig. 6h), whereas it is less pronounced in the other sites and clusters, likely due to the strong hybridization between O- and Ni-states in this region. Even in clusters 3 and 7, characterized by smaller O-Co distances, the peak is quite broad due to the shorter O-Ni separation (Fig. 6h, m).

The presence of Mn in the local environment leads to strongly hybridized states in the range $3-4$ eV and gives rise to sharp peaks. The O-PDOS decreases with increasing distance as seen in clusters 2 and 4 (Fig. 6g,i). This trend is confirmed in cluster 5 (Fig. 6j) as Mn-neighbors are available only to a limited number of sites with a median distance on the order of 2.8 Å (the larger confidence interval in Fig. 6d is due to the small number of samples in this cluster). Therefore, such a peak is either absent or very weak in the corresponding O-PDOS. Due to the presence of Mn, a second peak arises at 1 eV. The comparison between cluster 2 (see also cluster 1 in the SI) and cluster 5 emphasizes this point (Fig. 6g, j). Although Mn is present in clusters 3 and 4, this peak is not visible in the O-PDOS (Fig. 6h, i) due to the larger O-Mn distances. On the other hand, this peak is visible in cluster 6 characterized by shorter O-Mn separations (see Fig. 6k). It is clear from this discussion that structural similarities and in particular interatomic distances, related to orbital hybridization, are crucial to explain specific electronic-structure features.

**Fig. 7:**
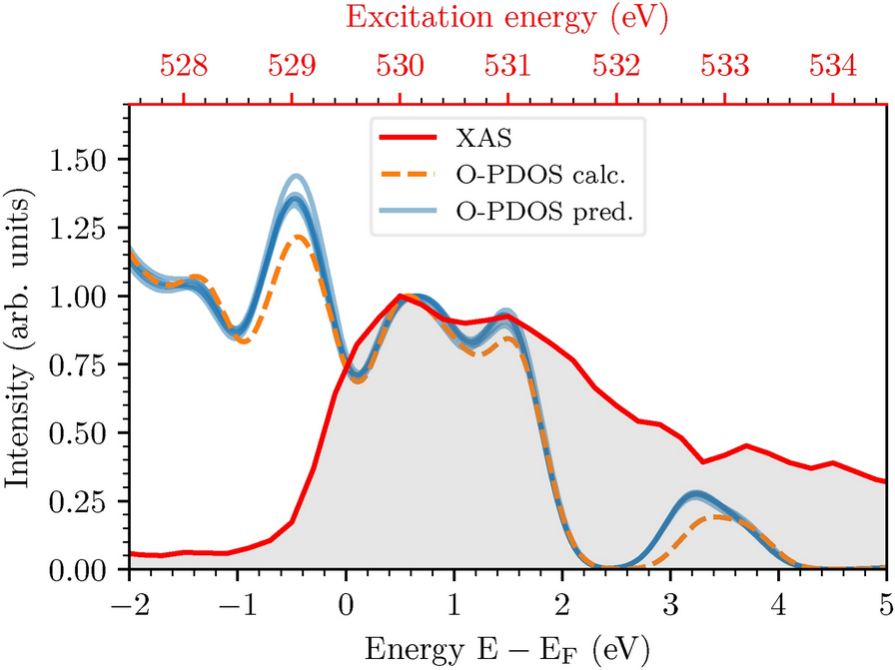
Experimental O *K*-edge PFY XAS data (red curve) of NCM-811 compared with *predicted* PDOS (blue curves) obtained by combining PDOS of individual oxygen clusters based on the cluster analysis and the *calculated* PDOS (orange dashed curve) being the result of the DFT calculation that best matches the measurement. The PDOS across all orbitals is used, similar to the cluster analysis, but we carefully checked that the considered region contains only O-p contributions. Both PDOS are energetically aligned and normalized around 0.5 eV (bottom *x*-axis offset at the Fermi level ($\hbox {E}_F$) obtained from the DFT calculations of the PDOS) or 530 eV (top *x*-axis, where corresponding excitation energies are reported) to the first peak of the measured XAS, and smoothed by a Gaussian broadening of 0.5 eV.

### PDOS prediction

The cluster analysis presented above can be used to assess the characteristics of the NCM-811 sample probed experimentally. This analysis is based on the correlation of O-p states representing the target of excitations probed by O *K*-edge XAS PFY extracted from the RIXS map^31^, see Fig. S7. O *K*-edge spectra include transitions from O 1*s* core levels to unoccupied states. The lower-energy peaks (529 - 535 eV) correspond to excitations to states hybridized with transition-metal 3*d* orbitals, while higher-energy peaks (535-543 eV) correspond to excitations to states hybridized with metal 4*s* and 4*p* orbitals^72^ (see Fig. S10 for an extended plot). The former arise from the strong hybridization between transition metal 3*d* and O 2*p* states, while the latter from weakly structured Ni 4*s*/4*p*-O 2*p* mixing.

To reconstruct the total O-*p* contributions to the PDOS that best match the experimental XAS, we apply a procedure combining the cluster coefficients (number of sites per cluster from Fig. 3b) and the mean PDOS per O-cluster. Considering all possible combinations of the coefficients $c_1 \ldots c_7$, where $c_i$ represents the number of O-sites in a given structure under the constraint $\sum _{i=1}^{7} c_i = 20$ (each structure contains 20 O-sites), we generate 230230 PDOS predictions. Since Mn and Co atoms are rarely present, or even absent, in the local environment of the O clusters (see Fig. 6a-f, upper panels), their relative occurrences provides insights into their local availability. The expected number of Mn-O and Co-O pairs for each prediction is calculated by a linear combination of these expectation values per cluster and the cluster coefficients. In the 28 considered structures, each Mn site has 6 or 7 O neighbors, while each Co site has 6. Because the structural space is limited, greater variation would be expected without these constraints.

Hence, we imposed: $4.5 \le \mathrm {N_{Co-O}} \le 7.5$ and $4.5 \le \mathrm {N_{Mn-O}} \le 8.5$, whereby $\mathrm {N_{Co-O}}$ and $\mathrm {N_{Mn-O}}$ indicate the expected number of Co-O pairs and Mn-O, respectively. These boundaries are chosen to reduce the number of predictions while maintaining sufficient flexibility to explore the influence of varying metal-oxygen pair numbers. This resulted in 3706 PDOS predictions. Among these, six entries exhibited nearly identical results (blue curves in Fig. 7), a consequence of the similarity of PDOS contributions between certain clusters and the inherent difficulty in defining a single optimal metric for comparison with the experimental data.

These predictions show excellent agreement with the experimental XAS data, indicating that the local environment of the O-sites is given by clusters 1, 3, 5, and 7 (see Fig. S9). These clusters are also present in structure 1, which corresponds to the best match with the measured data (Fig. 3 and SI for more details), although it experiences a redistribution of the number of sites per cluster. In the predictions, the number of sites is distributed more uniformly and less focused on cluster 7, indicating slightly larger average distances to the neighbors at higher indices.

Crucially, the DFT results accurately reproduce the XAS signal in the low-energy region ($0 - 2$ eV in Fig. 7). In particular, the simulations capture the double-peak structure suggested by the experimental data. This agreement extends beyond the energy of the maxima; the relative oscillator strengths of the two peaks are also accurately reproduced, with the lower-energy peak exhibiting a slightly larger intensity as in the experiment. At higher energies, above 2 eV, the PDOS drops and rises again with a maximum close to 3 eV. This feature can be associated with another peak in the experimental spectrum although the agreement is not as good as at lower energies. In particular, the cluster predictions (solid blue lines in Fig. 7) are closer to the experimental peak at 3 eV than the PDOS of best matching calculation (dashed orange curve).

While limitations of the adopted PBE functional could contribute to this slight discrepancy, some fundamental aspects should be taken into account. First, in the comparison between the XAS spectrum and the PDOS, it is important to keep in mind that the latter represents the density of available final states for the core-level transitions, while the XAS intensity reflects the transition probabilities between initial and final states. Therefore, even if the PDOS shows available states at higher energies, the corresponding transitions might have lower probabilities, leading to discrepancies with the intensity measured by XAS. This is especially relevant in complex systems like NMC-811, where multiple transitions can contribute to the spectrum. Furthermore, the alignment between experimental and calculated results at their onset magnifies the agreement in that energy range at the expense of higher-energy features. Finally, the intrinsic broadening of the experimental XAS spectrum, due to factors such as core-hole lifetime and instrumental resolution, becomes more significant at higher energies where the density of transitions increases. This broadening can obscure finer details in the spectrum, making a direct comparison with the calculations less straightforward.

Comparing our DFT calculations and especially the clustering-based predictions with previous results, we notice a good agreement with the findings presented in Refs.^23^ and^67^. However, in the PDOS of our best matching structure (Fig. 7 and Fig. S6), the oxygen peaks appear around 3.5 eV, *i.e.*, at slightly higher energies compared to those found by Dixit et al.^23^. These deviations can be related to the use of DFT+U or to the different stoichiometries of the considered structures, differing in particular in the relative Mn- and Ni-content.

Overall, the presented approach provides a powerful tool for interpreting XAS spectra and gaining a microscopic understanding of the local atomic and electronic structure in complex materials like NMC-811. This combined experimental-theoretical analysis offers a deeper understanding than what is available from the experiment alone, allowing us to connect the local atomic structure to macroscopic material properties. This information is crucial to better understand the behavior of these cathode materials in *operando* conditions and to optimize their design for enhanced performance.

## Summary and conclusions

In summary, we presented a high-throughput DFT analysis of the electronic-structure fingerprints on NCM-811. After the generation of candidate structures retrieved from data mining and the investigation of their structural similarities, we analyzed their atom-projected density of states. To efficiently address the intrinsic complexity of these systems, we adopted a clustering procedure to detect and interpret the signatures of site-specific PDOS contributions. This procedure offered a deep understanding of the impact of the local environment on the electronic properties of the system, unraveling the relation between structural and electronic properties. The analysis of the Ni PDOS enables the identification of the electronic fingerprints of different Ni oxidation states and the corresponding structural features of the local environments, such as the Jahn-Teller distortions of the octahedral Ni-O arrangement in the case of $\hbox {Ni}^{3+}$. Regarding the oxygen contributions to the PDOS, we could correlate specific features with the proximity to different transition-metal species. Through this analysis, we could predict the PDOS of an actual NCM-811 sample through the comparison with experimental X-ray spectroscopy data from the O *K*-edge, by decomposing the O *K*-edge XAS into linear combinations of the mean electronic features per cluster.

To conclude, the presented computational method is general and can be applied to investigate the correlation between structural and electronic properties in any complex material that is relevant for the interpretation of spectral fingerprints from XAS. This approach can be easily extended to other (site-specific) structural properties that impact the electronic structure. Instead of the PDOS adopted here, results of more advanced calculations, based for example on *ab initio* many-body perturbation theory^73–76^, can be employed. Beyond its computational efficiency, our method represents a viable and generalizable tool to complement experiments in the characterization of complex materials. These characteristics make it a valuable extension to existing data-driven approaches, particularly those relying on deep learning and massive datasets, for analyzing experimental spectroscopic data in conjunction with *ab initio* simulations^77–79^. Future developments including the integration with machine learning algorithms are expected to further enhance the capabilities of this numerical approach to address even more complex materials and configurations. While this study has been primarily motivated by the need to complement experimental studies based on X-ray absorption spectroscopy techniques with efficient computational methods to rationalize the fingerprints of NCM-811 samples and their degradation products, the perspective to enhance the developed approach with artificial intelligence algorithms can become particularly important to predict how the presence of defects, dopants, or additives affects the electronic structure of these systems. This knowledge is essential to design viable ways to enhance the energy storage performance of NCM-811 and related materials.

## Supplementary Information

Supplementary Information.

## Data Availability

The computational data produced in this work are available free of charge in the open-access repository Zenodo: 10.5281/zenodo.12600201.
