# Supplementary material for: Combinatorial ab initio calculations and core spectroscopy unravel the electronic structure of nickel cobalt manganese oxide
Source: Sci Rep. 2025 Feb 17;15:5816. doi: 10.1038/s41598-025-89283-8 (PMC11833067; doi:10.1038/s41598-025-89283-8)
Supplement: Supplementary file 1 — Supplementary Information. [file 41598_2025_89283_MOESM1_ESM.pdf]

SUPPORTING INFORMATION

Combinatorial *Ab Initio* Calculations and Core Spectroscopy Unravel the Electronic Structure of Nickel Cobalt Manganese Oxide

Timo Reents,[1] Elmar Kataev,[2] Daniel Duarte-Ruiz,[1] Regan G. Wilks,[2] Raul Garcia-Diez,[2] Marcus Bär,[2,3,4,5] and Caterina Cocchi[1,6,7]

[1]*Carl von Ossietzky Universität Oldenburg, Institute of Physics, Carl-von-Ossietzky Strasse 9, 26129 Oldenburg, Germany*

[2]*Department of Interface Design, Helmholtz-Zentrum Berlin für Materialien und Energie GmbH (HZB), Albert-Einstein Str. 15, 12489 Berlin, Germany*

[3]*Energy Materials In-Situ Laboratory Berlin (EMIL), HZB, Albert-Einstein Str. 15, 12489 Berlin, Germany*

[4]*Department of Chemistry and Pharmacy, Friedrich-Alexander-Universität Erlangen-Nürnberg (FAU), Egerlandstr. 3, 91058 Erlangen, Germany*

[5]*Dept. X-ray Spectroscopy at Interfaces of Thin Films, Helmholtz-Institute Erlangen-Nürnberg for Renewable Energy (HIERN), Albert-Einstein-Str. 15, 12489 Berlin, Germany*

[6]*Carl von Ossietzky Universität Oldenburg, Center for Nanoscale Dynamics, Carl-von-Ossietzky Strasse 9, 26129 Oldenburg, Germany*

[7]*Corresponding author: caterina.cocchi@uni-oldenburg.de*

(Dated: January 28, 2025)

I. STRUCTURAL ANALYSIS

A. Structural similarity initial structures

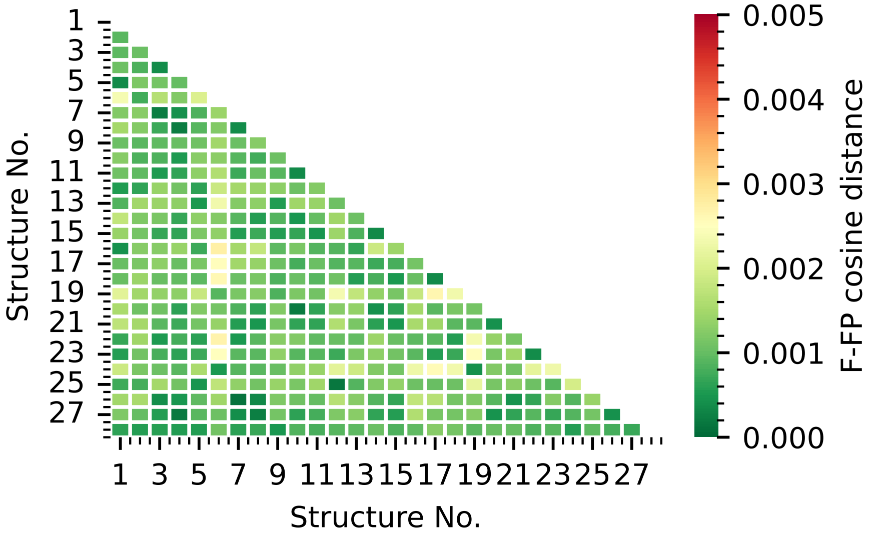

FIG. S1. Pairwise similarity between the different initial structures evaluated from the F-fingerprint and the cosine distance. (Note the smaller scale in comparison to the main text)

B. Energetic stability

TABLE S1. Energies per atom with respect to the most stable structure.

structure no.	energy $\frac{eV}{atom}$
18	0.0000
17	0.0008
23	0.0008
22	0.0012
5	0.0016
16	0.0016
1	0.0019
25	0.0020
9	0.0022
21	0.0022
13	0.0024
20	0.0025
8	0.0028
27	0.0029
7	0.0030
14	0.0030
28	0.0030
15	0.0033
10	0.0033
12	0.0038
26	0.0039
11	0.0041
4	0.0044
3	0.0058
2	0.0064
24	0.0067
19	0.0076
6	0.0077

II. CLUSTERING ANALYSIS

A. PDOS of O clusters with low contributions

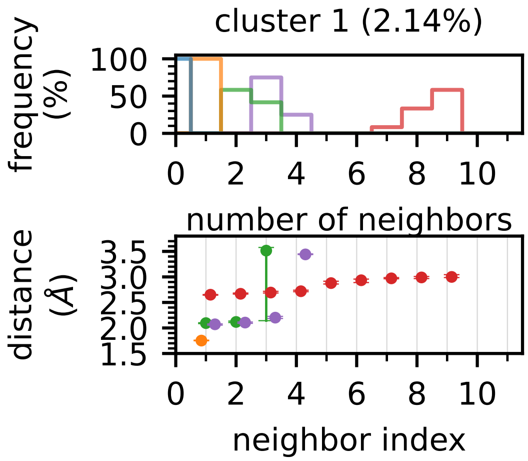

FIG. S2. Structural properties of the O PDOS cluster 1 contributing by 2.14%. The upper panel shows the number of atoms of a given species in the local environment of the indicated O-site. The lower panel represents the median of the distances to a given element with the error bars showing the 95%-confidence interval determined using bootstrapping. The distances are plotted over the neighbor index counting the ordered neighbors per species, where index 1 corresponds to the closest neighbor of a species. The data points are arbitrarily displaced around the actual integer values (indicated by the gray lines) to enhance visualization.

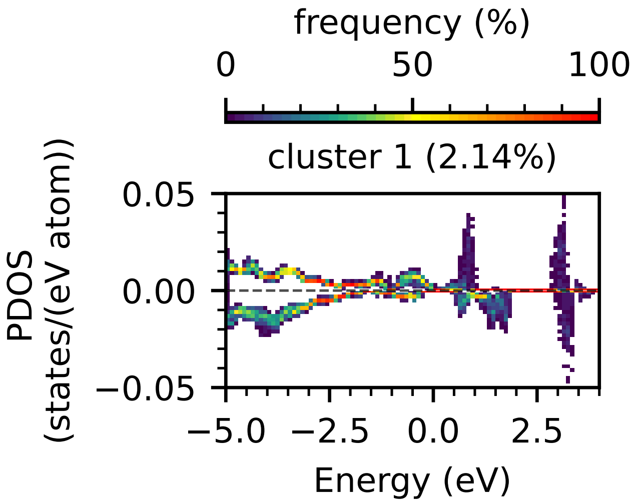

FIG. S3. PDOS contributions of the O-sites of the 28 NCM-811 structures belonging to cluster 1. Each site is considered separately whereby the spin-polarization was taken into account during the clustering (positive and negative values are associated with spin-up and spin-down contributions, respectively, and the Fermi energy is set to zero). The PDOS is represented as a 2D-histogram to allow statistical conclusions (the counts per cell are normalized by the number of sites so that each column sums up to 100% per spin channel) whereby the x-axis is discretized in steps of 0.05 eV. The relative amount of O atoms belonging to cluster 1 is indicated in the title.

1. Structural properties

TABLE S2. Number of neighbors of each species in the local environments of the O-sites. All results are normalized per neighboring element and cluster.

element	cluster<br>occurrences	1
Co	0	1.000
	1	-
Li	3	0.139
	4	0.861
	5	-
	6	-
Mn	0	-
	1	1.000
Ni	1	-
	2	0.194
	3	0.722
	4	0.056
	5	0.028
O	4	-
	5	-
	6	-
	7	0.194
	8	0.778
	9	0.028
	10	-
	11	-

TABLE S3. Median distances to the neighboring sites in the local environment of the O-sites: *element* indicates the species of the neighboring site and *index* represents the index of the sorted (in ascending order) neighboring sites per species. All results are given in Å and aggregated per neighboring element and cluster.

element	cluster<br>index	1
Co	1	-
Li	1	2.07
	2	2.11
	3	2.20
	4	3.44
	5	-
	6	-
Mn	1	1.75
Ni	1	2.10
	2	2.12
	3	3.52
	4	-
	5	-
O	1	2.65
	2	2.67
	3	2.69
	4	2.72
	5	2.88
	6	2.93
	7	2.97
	8	2.99
	9	3.00
	10	-
	11	-

B. Magnetic moments

Table S4 shows the mean and standard deviation of the magnetic moments per Ni-site and cluster. Those aggregated statistics highlight the relation between certain clusters and the different oxidation states, as indicated in the main text.

TABLE S4. Mean and standard deviation of the magnetic moments of Ni atoms in each cluster.

cluster	mean [μ_B]	standard [μ_B]
1	0.081	0.079
2	1.40	0.029
3	0.77	0.066
4	1.39	0.041

Table S5 presents the mean magnetic moment and the corresponding standard deviation per O-site and cluster. Clusters 3 and 5 show larger magnetic moments which can be seen in the PDOS as well (see main text, Fig. 6), due to the shift and the pronounced features in the spin-up channel below the Fermi level. The remaining clusters show smaller magnetic moments whereby the standard deviation is quite large, especially in clusters 4 and 7, in comparison to the corresponding mean.

TABLE S5. Mean and standard deviation of the magnetic moment for each O-site per cluster.

cluster	mean [μ_B]	standard [μ_B]
2	0.091	0.014
3	0.14	0.050
4	0.060	0.027
5	0.12	0.048
6	0.084	0.017
7	0.085	0.047

C. Coordination numbers to describe the structural properties of the clusters

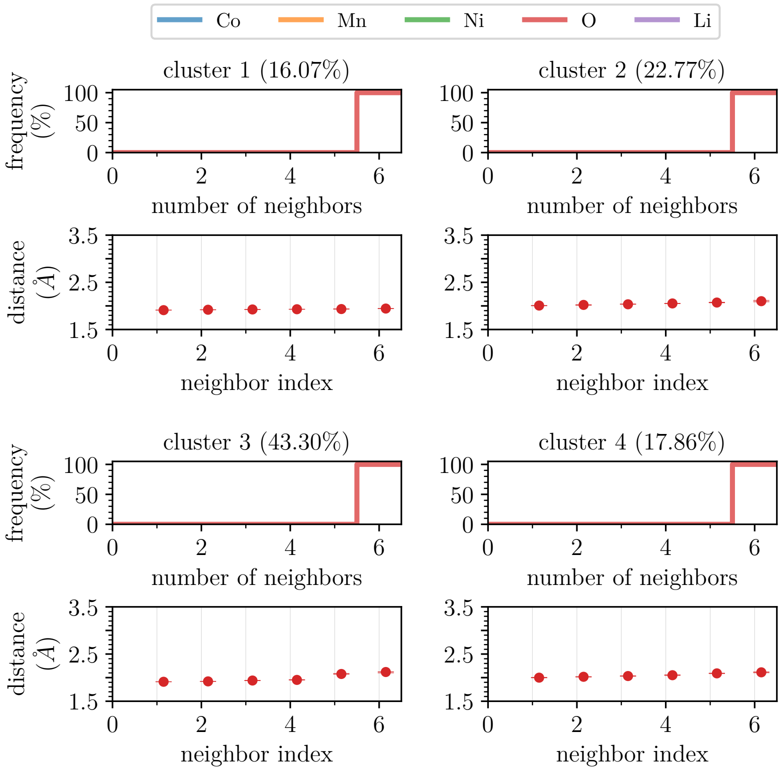

FIG. S4. Structural properties of the Ni-PDOS clusters (relative amounts given in parentheses). The subplot in the upper panel shows the distribution of coordination numbers of each Ni-site of a given neighboring species. The lower panel represents the median of the distances to a given element including the 95%-confidence interval calculated via bootstrapping and visualized as error bars. The distances are plotted over the neighbor-index (counting the ordered neighbors per species) with the index 1 corresponding to the closest neighbor of a species. The data points are arbitrarily displaced around the actual integer values (indicated by the gray lines) to enhance visualization.

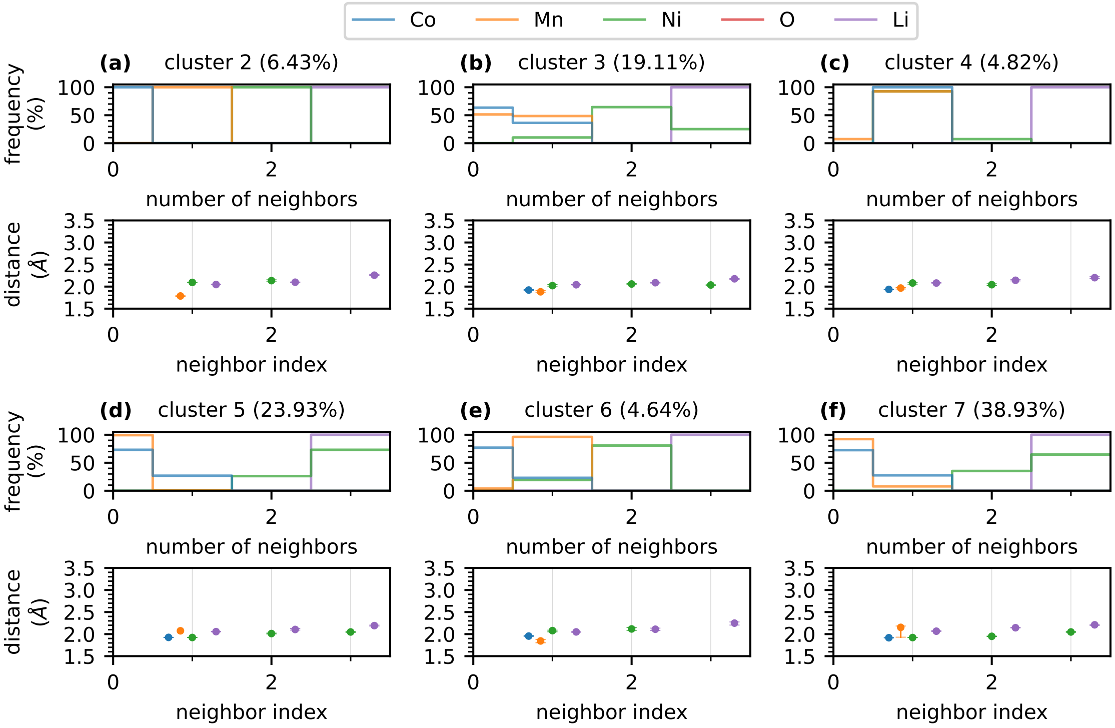

FIG. S5. Structural properties per O-PDOS cluster. The first subplot in each cluster shows the distribution of coordination numbers of each O-site of a given neighboring species. The lower panel represents the median of the distances to a given element including the 95%-confidence interval calculated via bootstrapping and visualized as errorbars. The distances are plotted over the neighbor-index (counting the ordered neighbors per species) whereby the index 1 corresponds to the closest neighbor of a species. The data points are arbitrarily displaced around the actual integer values (indicated by the gray lines) to enhance visualization. The relative amount of O atoms per cluster is mentioned in title.

Figs. S4 and S5 show the structural features determined via coordination environments (in contrast to the full Voronoi analysis presented in the main text). It should be noted that long-range effects are not included in this analysis.

D. PDOS of the best matching structure

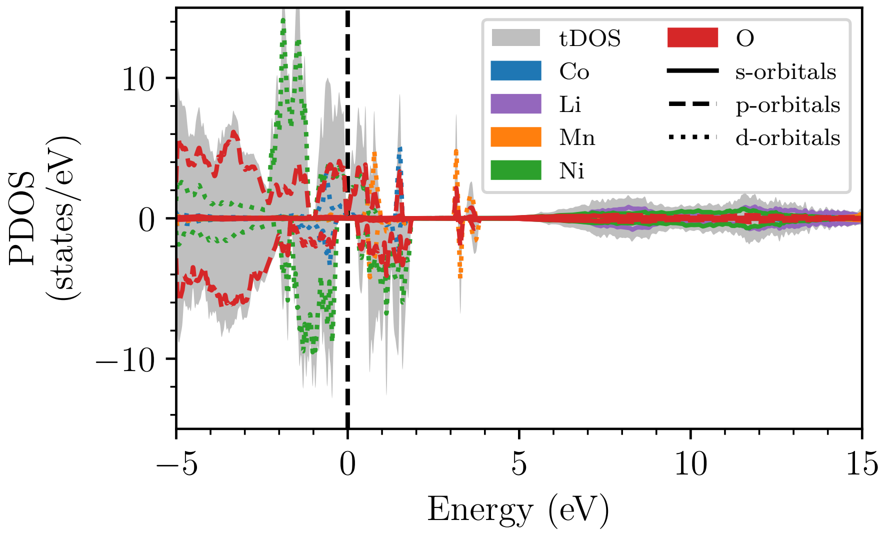

FIG. S6. PDOS of the best matching calculated structure (structure No. 1). The PDOS is summed per element. Positive and negative values correspond to spin-up and spin-down, respectively.

III. EXPERIMENTAL DATA

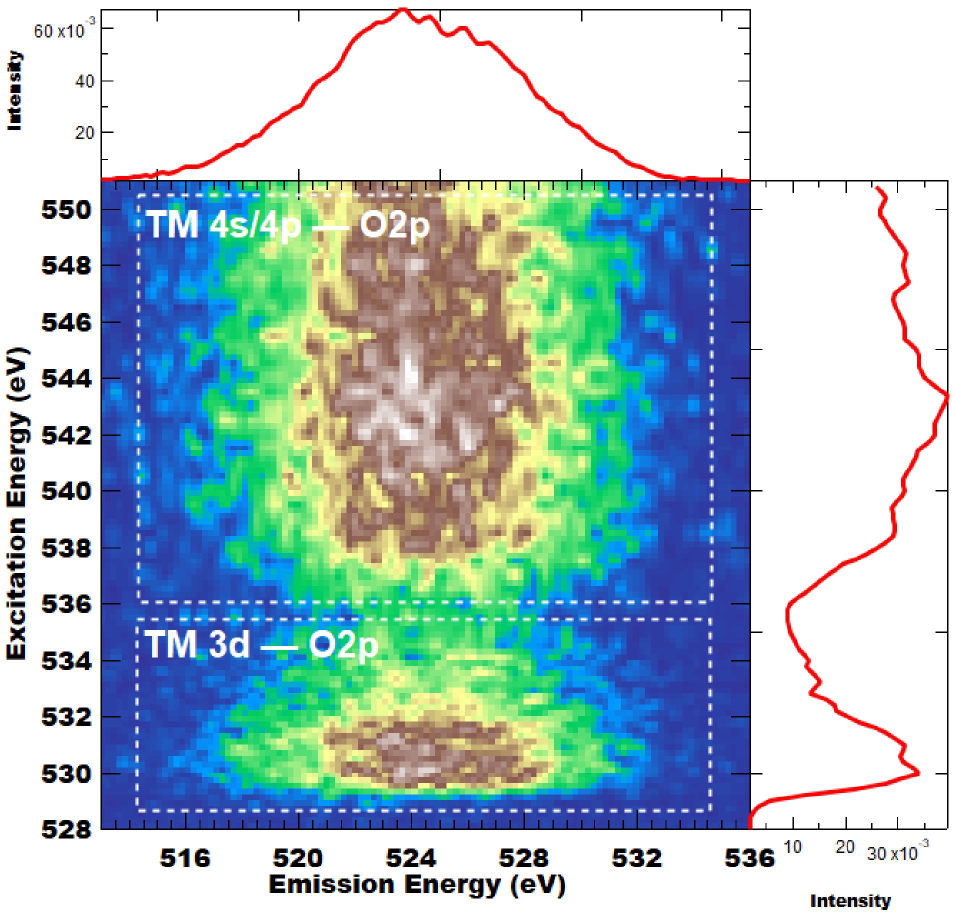

FIG. S7. Experimentally resonant inelastic X-ray scattering (RIXS) map of NCM-811 at the O K-edge. X-ray absorption (XAS) and emission (XES) signals, obtained by integrating the RIXS map in one direction, are shown on the right and top panels, respectively.

IV. COMPARISON OF CALCULATED PDOS AND EXPERIMENTAL XAS AND XES

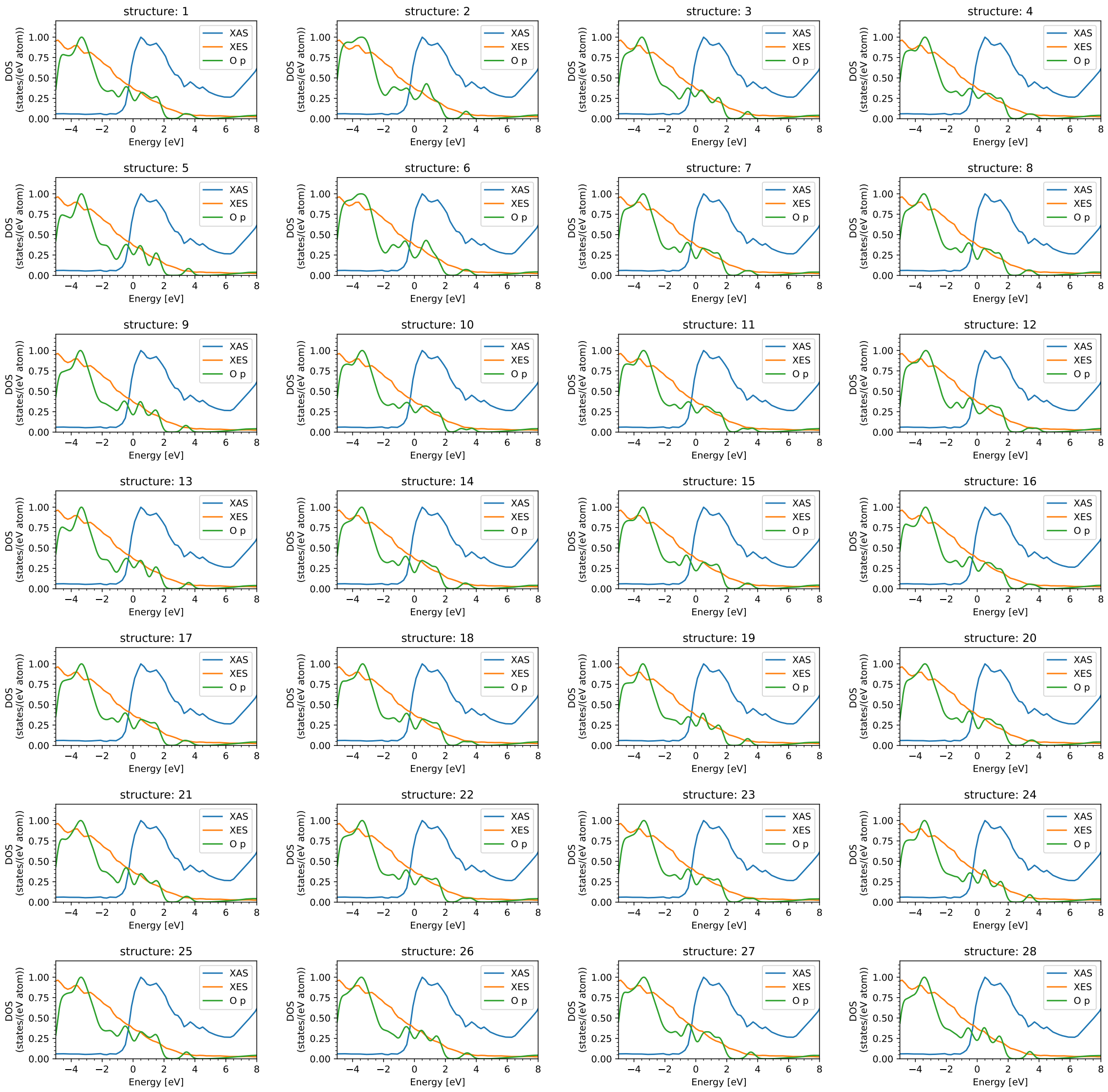

FIG. S8. Comparison of experimental XAS and XES data (extracted from RIXS measurements) of NCM-811 with calculated O-p PDOS for all 28 structural candidates. The calculated PDOS (summed over spin channels) is broadened by 0.5 eV and the Fermi level is shifted to 0 eV.

TABLE S6. Similarity values of the O-PDOS of each structural candidate and experimental XAS based on the χ^2-test. The values are sorted according to the highest similarity. Only the conduction band is used for the evaluation. The data are normalized to the peak between 0 and 0.5 eV.

structure no.	χ^2
1	117.96
10	118.23
11	118.47
4	118.59
23	120.50
16	120.77
18	120.78
27	121.14
20	121.46
22	122.12
17	122.60
12	122.81
25	123.99
21	124.33
7	126.48
15	127.68
8	128.43
26	130.60
14	132.17
13	135.33
3	135.95
9	140.61
5	142.09
28	143.15
24	145.02
19	146.23
2	148.44
6	149.33

TABLE S7. Similarity values of the O-PDOS of each structural candidate and the experimental XAS based on the metric proposed by Kuban *et al.* [1] and used in the analysis reported in the main text. The values are sorted according to the highest similarity. Only the conduction band is used for the evaluation. The data are normalized to the peak between 0 and 0.5 eV.

structure no.	DOS similarity metric
4	0.5570
23	0.5559
22	0.5505
1	0.5460
10	0.5444
11	0.5436
17	0.5421
18	0.5411
25	0.5367
15	0.5355
27	0.5351
20	0.5332
16	0.5318
12	0.5294
7	0.5269
8	0.5238
21	0.5051
14	0.5034
26	0.4944
3	0.4708
13	0.4704
9	0.4494
5	0.4456
28	0.4419
24	0.4294
19	0.4258
6	0.4242
2	0.4235

The χ^2-value and the DOS-similarity-metric are used to quantitatively determine the similarity between the O-p states of the different structural candidates and the experimental XAS data. The comparison is limited to the conduction region. Moreover, the O-p states are normalized to the peak between 0 and 0.5 eV. The two metrics yield similar results, with a few exceptions. According to these metrics, structures 1 and 4, showing similar shapes, are the best matches. Structure 1 is chosen by visual inspection as the best matching one plotted in Fig. 7 in the main text.

V. PDOS PREDICTION BASED ON CLUSTERING

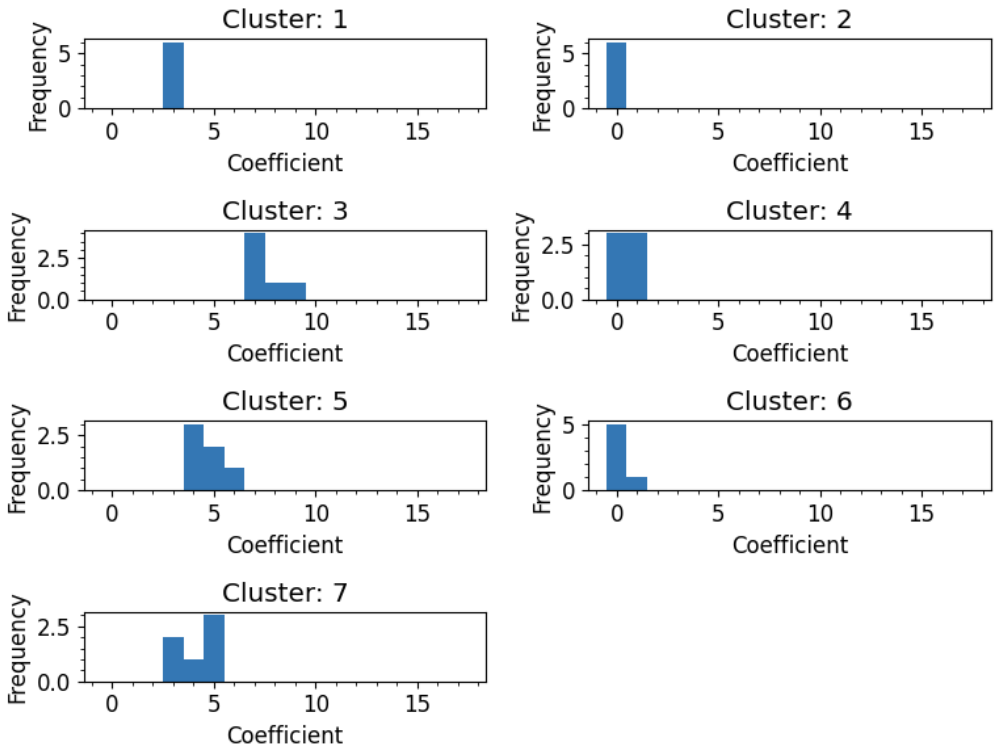

FIG. S9. Histograms showing the value of each cluster coefficient per O-cluster for the 6 best predictions.

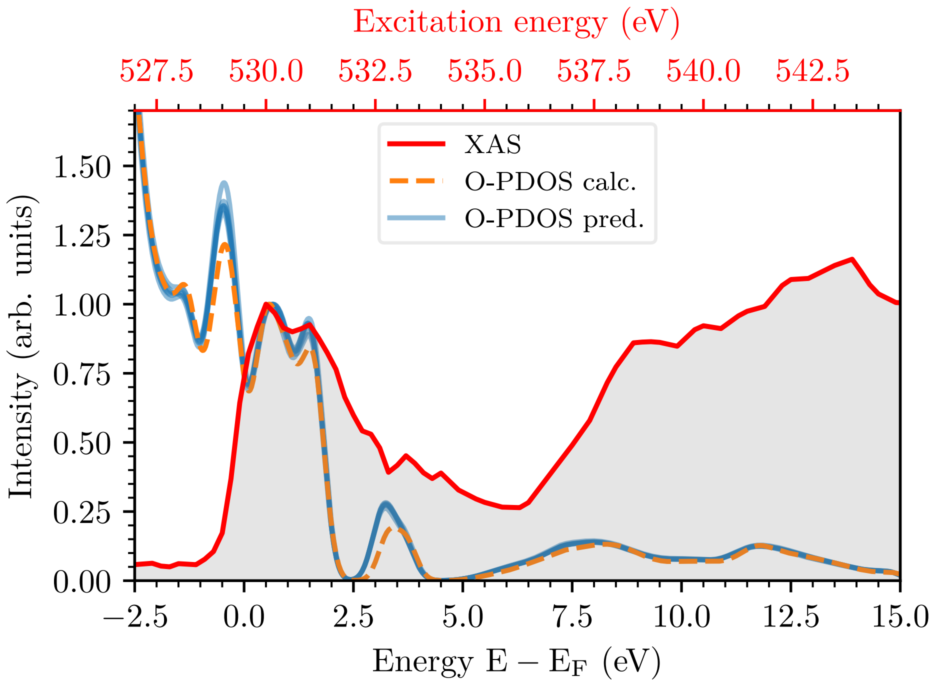

FIG. S10. Experimental O K-edge XAS data (red curve), extracted from RIXS measurements of NCM-811, compared with the best matching predictions for the O-PDOS based on the cluster analysis (blue) and the best matching DFT calculation (orange dashed line), covering a more extended energy range than Fig. 7 in the main text. In the region around 7.5 eV O-s contributions are present (not shown). The predicted and calculated PDOS are energetically aligned and normalized around 0.5 eV according to the scale of the bottom axis obtained from the DFT calculations and offset at the Fermi level (E_F), or 530 eV with respect to the top x-axis referred to the energy scale of the measured XAS. A Gaussian broadening of 0.5 eV is included to visualize the PDOS curves.

[1] M. Kuban, S. Rigamonti, M. Scheidgen, and C. Draxl, Sci. Data **9**, 646 (2022).
